# Impact of Q-fever fatigue syndrome on patients’ work status

**DOI:** 10.1093/occmed/kqaa166

**Published:** 2020-10-19

**Authors:** D F M Reukers, J A F van Loenhout, I Roof, T F Senden, S P Keijmel, C P Bleeker-Rovers, C H M van Jaarsveld, J L A Hautvast, K van der Velden

**Affiliations:** 1 Department of Primary and Community Care, Radboud University Medical Center, Radboud Institute for Health Sciences, Nijmegen, The Netherlands; 2 Center for Research on the Epidemiology of Disasters (CRED), Institute of Health and Society, Université Catholique de Louvain, Brussels, Belgium; 3 Department of Neurology, Radboud University Medical Center, Donders Institute for Brain Cognition and Behaviour, Nijmegen, The Netherlands; 4 Department of Internal Medicine, Radboud University Medical Center, Radboud Expertise Center for Q-fever, Nijmegen, The Netherlands

**Keywords:** Job satisfaction, Q-fever, QFS, Work Ability Index, working hours

## Abstract

**Background:**

Chronic illnesses can increase the risk of unemployment, but evidence on the specific impact of Q-fever fatigue syndrome (QFS) on work is lacking.

**Aims:**

The aim of this study was to describe and quantify the impact of QFS on work.

**Methods:**

Changes in work status from 1 year prior to 4 years after acute Q-fever infection of QFS patients were retrospectively collected with a self-report questionnaire measuring employment status and hours of paid work per week. In addition, information on work ability, job satisfaction and need for recovery after work was collected in 2016. Data were compared to participants from the general population.

**Results:**

The proportion of employed QFS patients from 1 year prior to 4 years after acute infection decreased from 78 to 41%, while remaining relatively constant in the general population (82 to 78%). Working QFS patients showed a decrease in mean hours of paid work from 35 to 22 h per week, which is significantly steeper compared to the general population (31–28 h per week) (*P* < 0.001). QFS patients showed a significantly lower work ability (*P* < 0.001), lower job satisfaction (*P* = 0.006) and greater need for recovery (*P* < 0.001) compared to the general population.

**Conclusions:**

The number of QFS patients with paid work decreased over the years, while patients who continue to work experience lower work ability, job satisfaction and increased need for recovery. Occupational physicians should be aware of the occurrence and severity of the impact of QFS on work, even after many years.

**Key learning points**

**What is already known about this subject:**

- Chronic illnesses can have an impact on work status and increase the risk of unemployment, but evidence on the specific impact of Q-fever fatigue syndrome on work is lacking.

**What this study adds:**

- Q-fever fatigue syndrome patients suffer from an impaired work status compared to the general population on different levels, as many become incapacitated or unemployed, decrease their working hours per week or show a significantly lower work ability and higher need for recovery.

**What impact this may have on practice or policy:**

- These results show that more research into targeted interventions to improve the work status of Q-fever fatigue syndrome patients may be warranted, as studies have shown that participation in work leads to better health outcomes.

## Introduction

The bacterium *Coxiella burnetii* can cause a zoonotic disease called Q-fever in humans. In 40% of acute Q-fever infections, patients present with a flu-like illness with clinical symptoms such as fever and headache [1], and sometimes pneumonia and hepatitis. Chronic fatigue develops in ~20% of patients with a symptomatic acute Q-fever infection, known as Q-fever fatigue syndrome (QFS) [2]. This illness is characterized by severe fatigue which may persist for up to 10 years [3].

Several Q-fever outbreaks have been documented worldwide, but the largest to date took place in the Netherlands and affected the general population with a total of 4107 cases of acute Q-fever notified between 2007 and 2011 [4]. Q-fever has long been considered an occupational illness, causing disease among farmers, veterinarians, abattoir and laboratory workers [5,6]. Therefore, in many countries vaccination of at-risk working populations is widely promoted. However, vaccination has not been implemented in the Netherlands. Earlier studies after the Dutch outbreak have shown that Q-fever infection can have an impact on work status. Approximately two out of five Q-fever patients were absent from work for longer than 1 month after acute infection [7]. If patients did resume their work activities, 9.3% could not function at the same level as before the Q-fever infection [7]. Another study concluded that one out of five previously working Q-fever patients showed a reduction in work participation 1 year after the initial infection [8]. Previous studies regarding Q-fever patients and work focused on short-time consequences, up to 2 years after acute infection. Evidence on the specific impact of long-term sequelae of infectious diseases, such as QFS, on work is lacking.

This is the first study to describe and quantify the impact of QFS on work, compared to the general population. The study focused on changes in employment status and number of working hours, from the year prior to Q-fever infection until 4 years after, and on current work ability, job satisfaction and need for recovery.

## Methods

Changes in work status covering a period of six yearly time points (1 year prior until 4 years after infection) were collected retrospectively. Data on current work status were collected cross-sectionally with a self-reported questionnaire.

QFS patients were recruited through physicians from the Radboud University Medical Center, which is the QFS expert centre in the Netherlands (see Table 1 for diagnostic criteria) [9].

**Table 1.** Diagnostic criteria for QFS [3]

Persisting fatigue for at least 6 months following acute Q-fever infection **AND** Laboratory-confirmed acute Q-fever, but no chronic Q-fever **AND** No existing somatic or psychiatric co-morbidity, which could explain the fatigue **AND** Fatigue causes significant limitations in daily functioning **AND** Complaints of fatigue were not present prior to the acute Q-fever infection or the complaints have since then clearly increased in severity.

A reference group consisting of people from the general population was recruited through two advertisements in local newspapers in a region outside the Q-fever epicentre and snowball sampling through included participants. For more details on recruitment of participants, see Reukers *et al.* [10].

Participants were asked to report their occupational history for the period January 2006 (i.e. 1 year prior to the start of the Q-fever epidemic in 2007) until 2016 in a self-report questionnaire. For every change in employment status, participants stated; (i) the start and end date; (ii) being in paid work or not; (iii) the number of paid working hours; (iv) being fully or partially occupationally incapacitated; and (v) type of occupation or reason for not working. Informal work (e.g. homemaker, student) was considered as ‘non-employed’. The mean number of hours of paid work per week was calculated for every time point. We hypothesized that patients might change jobs to adjust to their capacities, thereby preventing becoming unemployed or incapacitated. As a proxy for a change in job complexity, the occupational skill level was determined for each participant on each time point, based on type of occupation recorded, according to the International Standard Classification of Occupation (ISCO) [11]. Skill level 1 involves simple and routine tasks (e.g. office cleaners, garden labourers or kitchen assistants). Skill level 2 typically involves operating machinery or electrical equipment and requires relatively advanced literacy and numeracy skills (e.g. bus drivers, hairdressers or electricians). Skill level 3 involves complex technical and practical tasks requiring a high level of literacy and numeracy (e.g. legal secretaries, medical laboratory or computer technicians). While skill level 4 involves tasks with complex problem-solving and decision-making skills in a specialized field (e.g. medical professionals, secondary school teachers or civil engineers).

The following three work-related instruments were only reported by participants who had paid work at the time of filling out the questionnaire: (i) Work ability was measured using the Work Ability Index (WAI) [12]. (ii) General satisfaction with the current occupation was measured with the one-item job satisfaction scale (range 1–5)[13]. (iii) Work-related need for recovery was assessed with the need for recovery scale from the Questionnaire Experience and Evaluation of Work (VBBA) [14].

Data regarding age, gender, educational level, co-morbidity and date of Q-fever infection were also collected. Educational level was divided into low, moderate and high education [10]. Participants receiving treatment or regular health exams for any illness other than QFS were classified as having one or more (co-)morbidities. Conditions, such as hypertension, allergies or obesity, causing an increased risk of disease, but not being debilitating by themselves were not considered co-morbidities.

Data were analysed with SPSS, version 22. Demographic characteristics of QFS patients and persons from the general population were compared using ANOVA (for continuous variables) or chi-square tests (for categorical variables). To assess the impact over time, six yearly time points relative to the estimated date of acute Q-fever infection were selected. These time points ranged from the year prior up to 4 years after acute infection. In the general population, the mean date of acute Q-fever infection reported by the QFS patients was imputed to define the same six yearly time points (i.e. 20 August 2009).

**Figure 1.**
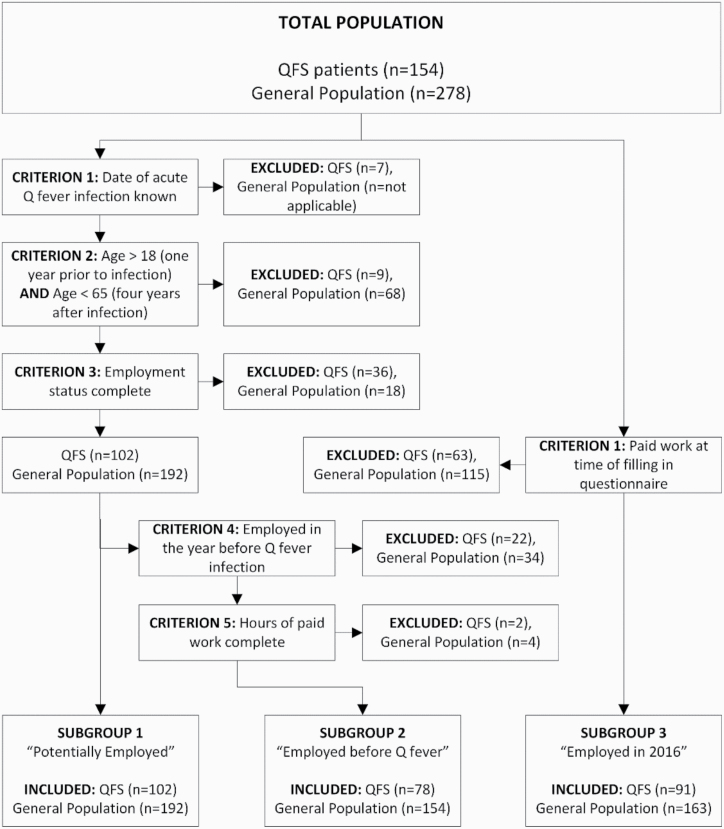
Flowchart displaying the definition of subgroups.

The following three subgroups were defined (Figure 1): subgroup 1 included participants who could potentially have had paid work according to their age and only participants with complete data on all time points were included; subgroup 2 included all persons from subgroup 1 who had paid work prior to the acute infection with complete data on all time points; subgroup 3 included participants who had paid work at the time of filling out the questionnaire in 2016, which is between 5 and 9 years after acute infection.

The proportion of participants in each category of employment status was calculated for each time point and study group. These proportions were compared over time within and between the study groups using chi-square tests. For the hours of paid work per week, repeated measures ANOVA was used to test whether there was a difference between the QFS patients and the general population across time points and in slope corrected for possible confounders (age, gender, educational level and co-morbidity). The skill level was compared between 1 year prior to acute infection and the last reported type of occupation between 2 and 4 years after acute infection. Scores on work ability, job satisfaction and need for recovery were analysed with an independent samples *T*-test to compare QFS patients with the general population and multivariate regression analysis to correct for possible confounders (age, gender, educational level and co-morbidity).

Ethical approval for the study protocol was obtained from the Medical Ethical Review Board of the region Arnhem-Nijmegen (NL55961.091.15). All participants gave written informed consent. Participants from the general population received a 10 Euro gift card after participation.

## Results

In total, 251 QFS patients were eligible, of which 154 (61%) completed the questionnaire. Three hundred thirty-one persons from the general population responded to the advertisement of which 278 (84%) completed the questionnaire. No statistically significant differences in age and gender distributions were found between responders and non-responders in both groups separately (data not shown). The characteristics of the two study groups for all participants and for every employment subgroup are presented in Table 2. QFS patients were significantly younger than the general population (48 versus 53 years, respectively, *P* = 0.001), but differences were not significant in any of the subgroups. In all the subgroups and the total group of participants, the general population showed a larger proportion of higher educated individuals compared to the QFS patients (range 57–60% compared to 39–44%, respectively). In subgroup 2 and subgroup 3, QFS patients showed a significant higher proportion of self-employment compared to the general population (17 and 24% compared to 8 and 12%, *P* = 0.039 and *P* = 0.009) (Table 2).

**Table 2.** Demographic and health characteristics per study group and proportion of self-employment in QFS patients compared to the general population

		QFS	General population	*P*-value
All participants		*n* = 154	*n* = 278	
Age	Mean (SD)	48 (12.1)	53 (15.4)	0.001
Gender				
Male	*n* (%)	67 (44)	142 (51)	0.131
Educational level				<0.001
Low	*n* (%)	21 (14)	22 (8)	
Moderate	*n* (%)	73 (47)	91 (33)	
High	*n* (%)	60 (39)	165 (59)	
(Co-)morbidity^a^	*n* (%)	59 (38)	93 (33)	0.311
Subgroup 1 Potentially employed		*n* = 102	*n* = 192	
Age	Mean (SD)	49 (10)	50 (12)	0.659
Gender				
Male	*n* (%)	45 (44)	85 (44)	0.980
Educational level				0.006
Low	*n* (%)	16 (16)	14 (7)	
Moderate	*n* (%)	45 (44)	67 (35)	
High	*n* (%)	41 (40)	111 (58)	
(Co-)morbidity^a^	*n* (%)	40 (39)	57 (30)	0.098
Subgroup 2 Employed before Q-fever		*n* = 78	*n* = 154	
Age	Mean (SD)	50 (10.2)	50 (11.0)	0.636
Gender				
Male	*n* (%)	34 (44)	70 (46)	0.787
Educational level				0.036
Low	*n* (%)	12 (15)	11 (7)	
Moderate	*n* (%)	34 (44)	56 (36)	
High	*n* (%)	32 (41)	87 (57)	
(Co-)morbidity^a^	*n* (%)	30 (39)	43 (28)	0.102
Self-employed prior to Q-fever	*n* (%)	13 (17)	12 (8)	0.039
ISCO Skill level^b^				0.740
Skill level 1	*n* (%)	2 (3)	3 (2)	
Skill level 2	*n* (%)	32 (47)	62 (44)	
Skill level 3	*n* (%)	22 (32)	41 (29)	
Skill level 4	*n* (%)	12 (18)	34 (24)	
Subgroup 3 Employed in 2016		*n* = 91	*n* = 163	
Age	Mean (SD)	46 (11.7)	47 (13.6)	0.339
Gender				
Male	*n* (%)	43 (47)	77 (47)	0.998
Educational level				0.049
Low	*n* (%)	8 (9)	8 (5)	
Moderate	*n* (%)	43 (47)	58 (36)	
High	*n* (%)	40 (44)	97 (60)	
(Co-)morbidity^a^	*n* (%)	28 (31)	42 (26)	0.392
Self-employed in 2016	*n* (%)	22 (24)	19 (12)	0.009

^a^One or more (co-)morbidities, other than QFS.

^b^10 QFS patients and 14 participants from the general population had a missing skill level.

**Figure 2.**
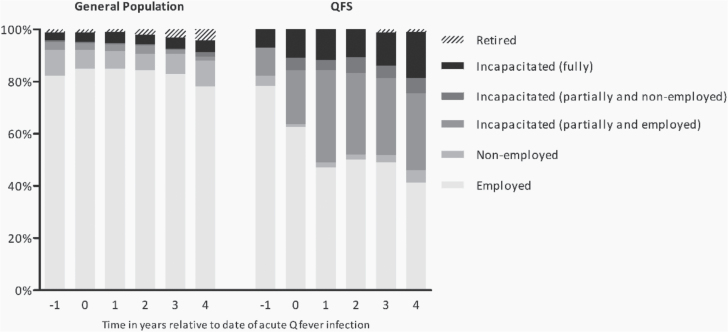
Bar chart representing the proportion of participants in every employment status category per year relative to the date of acute Q-fever infection for QFS patients and relative to 20 August 2009 for the general population (i.e. the average date of Q-fever onset for all QFS patients).

Criteria for potentially employed participants were met in 102 (66%) QFS patients and 192 (69%) persons from the general population (Figure 1). In the year prior to acute infection, 78% of the QFS patients in this subgroup were employed (Figure 2), which is comparable to the employed proportion in the general population (82%, *P* = 0.317). Four years after acute infection, the proportion employed in the QFS patients decreased to 41%, while remaining relatively constant in the general population (78%). The proportion fully incapacitated QFS patients in the year prior to acute infection (7%) was comparable to the proportion fully incapacitated in the general population (3%, *P* = 0.138). The proportion partially incapacitated (either with or without being employed) at 1 year prior to acute infection was significantly higher in QFS patients compared to the general population (11% versus 4%, *P* = 0.015). Figure 2 shows an increasing trend over time for the proportion partially incapacitated (either with or without being employed) QFS patients (11% 1 year prior, 35% 4 years after acute infection, *P* < 0.001), while the trend for fully incapacitated patients was not significant. Within the general population, the proportion fully or partially incapacitated did not show any increasing or decreasing trend over time.

**Figure 3.**
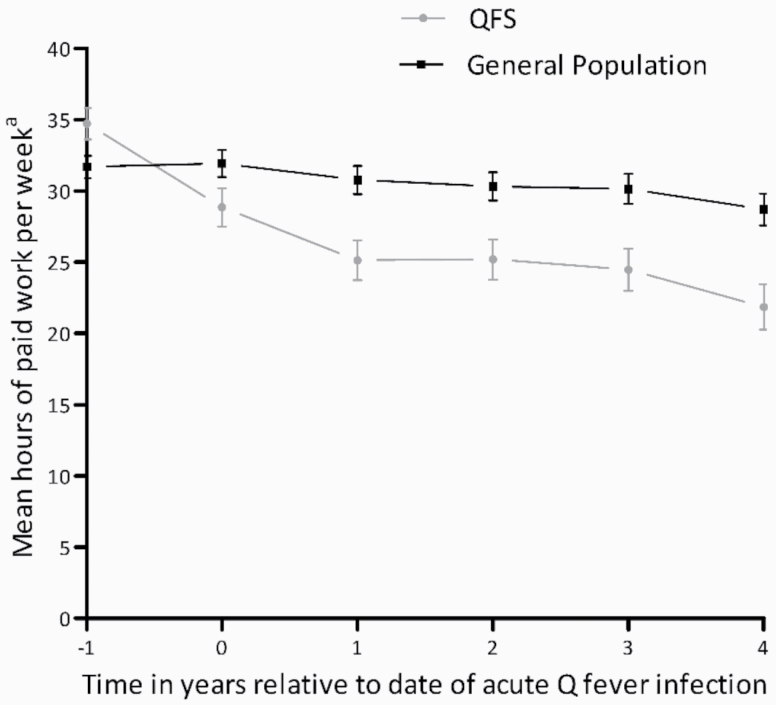
Mean hours of paid work per week (with 95% CIs) as estimated with repeated measures ANOVA for QFS patients and the general population per year relative to the date of acute Q-fever infection and relative to 20 August 2009 for the general population (i.e. the average date of Q-fever onset for all QFS patients). ^a^Corrected for age, gender and education level.

The subgroup ‘Employed before Q-fever’ consisted of 78 (51%) QFS patients and 154 (55%) participants from the general population (Figure 1). One year prior to infection, the QFS patients in subgroup 2 worked on average 35 h per week compared to 22 h 4 years after acute infection. The general population showed a decrease from 31 h 1 year prior to 28 h 4 years after the imputed date of infection. Results from the repeated measures ANOVA showed a significant decrease in hours per week over time in the total population (*P* < 0.001) (Figure 3). In this analysis, a significant interaction was found between time and study group; the decrease in the mean hours per week is significantly steeper in QFS patients compared to the general population (*P* < 0.001).

There were no relevant changes in skill level over time in both QFS patients and the general population (data not shown). Furthermore, the distribution of the skill levels prior to acute Q-fever infection was not significantly different between QFS patients and the general population (*P* = 0.740, Table 2).

Of the total population, 91 (59%) QFS patients and 163 (59%) persons from the general population were employed at the time of filling out the questionnaire (Figure 1). For the QFS patients this was on average 6.7 years after acute Q-fever infection. Analysis of the work-related measures in the population employed in 2016 showed that, after correction for confounders, QFS patients scored −3.56 [95% confidence interval (CI): −4.42; −2.70] points lower on the WAI compared to the general population (Table 3). Also, the scores on the one-item work ability (−2.4 [95% CI: −2.9; −1.9]) and job satisfaction (−0.4 [95% CI: −0.6; −0.1]) were significantly lower in QFS patients. The analysis showed that QFS patients employed in 2016 had a significantly higher need for recovery compared to the general population (44.64 [95% CI: 37.31; 51.96]).

**Table 3.** *T*-test and multivariate linear regression analysis of work ability, job satisfaction and need for recovery scores with unstandardized *B*-values and 95% CIs corrected for age, gender, educational level and (co-)morbidity in subgroup 3

		QFS	General population	*P*-value
		*n* = 91	*n* = 163	
ANOVA				
Work ability^a^	Mean (SD)	5.7 (2.3)	8.1 (1.7)	<0.001
WAI^a,b^	Mean (SD)	22.9 (3.9)	26.4 (3.0)	<0.001
Job satisfaction^b^	Mean (SD)	3.7 (0.9)	4.1 (1.0)	0.008
Need for recovery^b,c^	Mean (SD)	69.9 (29.2)	25.1 (26.9)	<0.001
Increased need for recovery^b,c^	*n* (%)	69 (77)	29 (18)	<0.001
Multivariate linear regression				
Work ability	*B* (95% CI)	−2.4 (−2.9; −1.9)	Ref	<0.001
WAI^a^	*B* (95% CI)	−3.6 (−4.4; −2.7)	Ref	<0.001
Job satisfaction^a^	*B* (95% CI)	−0.4 (−0.6; −0.1)	Ref	0.006
Need for recovery^a^	*B* (95% CI)	44.6 (37.3; 52.0)	Ref	<0.001

Higher scores indicate a higher work ability, job satisfaction and need for recovery.

^a^The WAI is composed of two parameters: a one-item work ability in which patients rate their current work ability on a scale of 1–10, with 10 representing the best period in their working life, and a composite work ability score (range 5–36) of multiple questions related to physical and psychological demands of work and possible limitations experienced during job practice.

^b^One missing value in the group of QFS patients.

^c^This scale is comprised of 11 dichotomous items to quantify difficulties participants experience in recovering from work. The scores on the individual items of this instrument were added and transformed into one score with a range from 0 to 100. A cut-off value of 54 was used to dichotomize the scale into categories of a normal and increased need for recovery.

## Discussion

This is the first study showing that QFS can have a significant impact on work. Among QFS patients, increasing numbers of incapacitated, a decrease in working hours per week, lower work ability, lower job satisfaction and higher work-related need for recovery was observed over time.

This study had several limitations. All data were self-reported. For the occupational history, it is highly likely that recall bias occurred in both QFS patients and controls. Studies on employment recall have shown that the accuracy can relate to the salience and complexity of the employment history, the length of the recall period and the presence of an important time-anchoring life event (such as marriage, child birth, etc.) [15–18]. However, these factors are not specifically related to one of these groups, so there is no reason to believe the amount of recall bias would differ considerably between groups. Although subjects with an incomplete questionnaire were contacted by telephone, this was not feasible for the occupational history part, as this was too complex. When analysing these data, only participants with complete data on all time points were included, which might have introduced a selection bias, although there was no significant difference in education level or employment status at the time of filling in the questionnaire between excluded and included participants. The selection for the potentially employed group was based on an age range of 18–65 years old. However, not everyone at age 18 is employed, and some people retire before age 65, as was seen in Figure 2. Despite these limitations, we feel this was the most exact way to present potentially employed individuals. Furthermore, the general population included a higher proportion of persons with higher education compared to the QFS patients. However, educational level was included in the analysis as a potential confounder and did not appear to be of influence. Also, the reference group was self-selected, which might have introduced some bias. Some participants might be more likely to participate for reasons that are correlated with current employment, for example unemployed or part-time working participants might have had more time to participate. However, this might have applied as well for the QFS population. Furthermore, we were interested in the work status 5–10 years ago, while the self-selection bias is related to the current situation and not necessarily to employment status in the past. Lastly, seronegativity for Q-fever was not tested in participants from the general population. It is possible that these participants had an asymptomatic or subclinical Q-fever infection. However, it is highly unlikely that this group included undiagnosed QFS patients.

The employment rate in QFS patients of 41% 4 years after infection is very similar to the employment rate of 43% of rheumatoid arthritis patients of working age with a mean disease duration of 4.3 years, as found in a Dutch study [19]. In this study, employment rate was defined in a similar way as in our study. A review regarding the prognosis of chronic fatigue syndrome, from 1 to 30 years after onset of symptoms, found a range in the work disability rate of 15–52% using the outcomes of several studies [20]. In a study regarding fatigue at work among employees from the Dutch general population between 18 and 65 years of age, fatigue was found to be a strong predictor of work disability [21]. Since fatigue is a highly characteristic symptom for QFS patients, this might explain the high rate of incapacitated patients in our study. Also, the proportion of self-employment was higher in QFS patients compared to the general population, which might have had an additional impact on the employment rate and the number of hours of paid work overtime. However, we did not identify any studies on whether self-employment poses a lower or higher risk on employment rate or incapacitation. Results also showed that a higher proportion of QFS patients were partially incapacitated before acute infection compared to the general population, but more research is needed to explain this difference.

The average number of 22 working hours per week after 4 years in the QFS group is low compared to the national average of 29 working hours in 2015 [22]. De Boer *et al.* studied the work status of inflammatory bowel disease patients and found a mean number of 31 working hours per week [23]. Van Loenhout *et al.* showed a reduction in working hours 1 year after infection of 50% and 40% in, respectively, Q-fever and Legionnaires’ disease patients [8]. In our study, QFS patients had a decrease of 28% in the mean hours of paid work 1 year after Q-fever infection compared to 1 year prior. Although a higher impact of QFS on working hours in comparison with Q-fever and Legionnaires’ disease was expected, a decrease of 28% in the hours of paid work is still a major impact. There were no relevant changes in skill level over time in either QFS patients or the general population.

Studies have shown that patients with chronic health problems can have decreased work ability and may leave the work force [24–27]. This study showed that QFS patients who continue to have paid work, experience lower work ability and job satisfaction, and increased need for recovery. Conversely, there is increasing evidence that loss of work and unemployment are strongly associated with poor physical and mental health [28], and that re-employment leads to an improved health and reduced psychological distress [29,30]. As QFS patients may benefit from re-entering the work force, future research should focus on identifying factors associated with an impaired work status and develop targeted interventions to enhance the work status of these patients.

Occupational physicians should be aware of the severity of the impact of QFS on work, even after many years. To provide better guidance, future research should focus on identifying factors explaining differences in work ability, work incapacity and employment between QFS patients and the general population. Research should also be directed to development of targeted interventions to retain and/or regain work ability and work.

## Funding

This work was supported by Stichting Q-support (AMPHI150114-00).
